# Campbell Standards: Modernizing Campbell's Methodologic Expectations for Campbell Collaboration Intervention Reviews (MECCIR)

**DOI:** 10.1002/cl2.1445

**Published:** 2024-10-06

**Authors:** Ariel M. Aloe, Omar Dewidar, Emily A. Hennessy, Terri Pigott, Gavin Stewart, Vivian Welch, David B. Wilson, Campbell MECCIR Working Group

**Affiliations:** ^1^ Educational Measurement and Statistics The University of Iowa Iowa City Iowa USA; ^2^ Temerty Faculty of Medicine University of Toronto Toronto Ontario Canada; ^3^ Department of Psychiatry, Massachusetts General Hospital Recovery Research Institute Boston Massachusetts USA; ^4^ College of Education and Human Development Georgia State University Atlanta Georgia USA; ^5^ University of Newcastle upon Tyne Newcastle upon Tyne UK; ^6^ Bruyere Research Institute Ottawa Ontario Canada; ^7^ Campbell Collaboration Ottawa Canada; ^8^ Criminology, Law and Society George Mason University Fairfax Virginia USA

**Keywords:** methodology, reporting guidelines, systematic review

## Abstract

**Introduction:**

The authors formed a small working group to modernize the Methodological Expectations for Campbell Collaboration Intervention Reviews (MECCIR). We reviewed comments and feedback from editors, peer reviewers of Campbell submissions, and authors; for example, that the Campbell MECCIR was long and some of the items in the reporting and conduct checklists were difficult to cross‐reference. We also wanted to make the checklist more relevant for reviews of associations or risk factors and other quantitative non‐intervention review types, which we welcome in Campbell. Thus, our aim was to develop a shorter, more holistic guidance and checklist of Campbell Standards, encompassing both conduct and reporting of these standards within the same checklist.

**Methods:**

Our updated Campbell Standards will be a living document. To develop this first iteration, we invited Campbell members to join a virtual working group; we sought experience in conducting Campbell systematic reviews and in conducting methods editor reviews for Campbell. We aligned the items from the MECCIR for conduct and reporting, then compared the principles of conduct that apply across review types to Preferred Reporting Items for Systematic Reviews and Meta‐Analyses (PRISMA)‐literature search extension (S) and PRISMA‐2020 reporting standards. We discussed each section with the aim of developing a parsimonious checklist with explanatory guidance while avoiding losing important concepts that are relevant to all types of reviews. We held nine meetings to discuss each section in detail between September 2022 and March 2023. We circulated this initial checklist and guidance to all Campbell editors, methods editors, information specialists and co‐chairs to seek their feedback. All feedback was discussed by the working group and incorporated to the Standards or, if not incorporated, a formal response was returned about the rationale for why the feedback was not incorporated.

**Campbell Policy:**

The guidance includes seven main sections with 35 items multifaceted but distinct concepts that authors must adhere to when conducting Campbell reviews. Authors and reviewers must be mindful that multiple factors need to be assessed for each item. According to the Campbell Standards, the reporting of Campbell reviews must adhere to appropriate PRISMA reporting guidelines(s) such as PRISMA‐2020.

**How to Use:**

The editorial board recommends authors use the checklist during their work in formulating their protocol, carrying out their review, and reporting it. Authors will be asked to submit a completed checklist with their submission. We plan to develop an online tool to facilitate use of the form by author teams and those reviewing submissions.

**Providing Feedback:**

We invite the scientific community to provide their comments using this *anonymous google form*.

**Plan for Updating:**

We will update the Campbell Standards periodically in light of new evidence.

## 1 INTRODUCTION

Methodological Expectations of Campbell Collaboration Intervention Reviews (MECCIR) constitute the standards for the conduct and reporting of Campbell systematic review of intervention reviews. These standards were initially adapted from the Cochrane MECIR conduct and reporting standards developed by the Cochrane MECIR team in 2014.

Since the development of the MECCIR, there have been significant improvements in methodological quality and completeness of reporting of reviews. We recently evaluated the quality of Campbell Systematic Reviews of interventions published between 2018 and 2022 and found 10% of the reviews were critically low quality and 29% low quality, using A MeaSurement Tool to Assess systematic Reviews (AMSTAR2). This suggests that either the current methodological expectations are not being implemented well or not sufficient to ensure adequate quality, or both. In addition, feedback from the authors and peer reviewers of Campbell Reviews and editorial board members suggest that the checklist is burdensome to complete due to the extensive list of over 130 reporting and conduct items. Thus, we decided to modernize and shorten MECCIR to focus more broadly on understanding of Campbell Standards, including guidance and a checklist that aims to harmonize conduct and reporting standards.

## 2 DEVELOPMENT

Our updated Campbell Standards will be a living document that will be updated periodically per the scientific community's feedback. To develop this first iteration, we established a working group composed of Campbell editorial board members who were experienced in conducting Campbell systematic reviews. We conducted a series of nine meetings (from September 2022 and March 2023) reviewing the 2014 MECCIR standards and identified seven core methodological aspects of reviews. Within each section, we iteratively developed items that describe essential concepts across review types, while harmonizing conduct and reporting standards. Subsequently, we cross‐referenced the updated MECCIR with (Preferred Reporting Items for Systematic Reviews and Meta‐Analyses) PRISMA‐2020 guidance, and PRISMA‐literature search extension (S) to ensure that the guidance adheres to current international reporting standards. We shared the first draft of the guidance with all the Campbell editors, methods editors and cochairs of the coordinating groups to obtain feedback that reflects divergent views and expertise and asked for feedback from June 2023 to August 2023. The MECCIR Working Group collated the responses to produce the full set of updated standards reported here.

## 3 IMPLEMENTATION OF THE STANDARDS

Each Campbell review should meet these Campbell Standards. All the items provided in the checklist are to be interpreted by authors as mandatory for a new protocol or review to be published in Campbell Systematic Reviews. It is important to note that some Campbell reviews are not focused on intervention effects, instead examining correlations or diagnostic test accuracy but may also synthesize research that informs policy decisions. Thus, we updated the checklist to cover the core methodological concepts that would be required in any high‐quality review of research and be appropriate regardless of the topic. Nonetheless, certain decisions on methods may need to be made depending on the research question and the type of data being synthesized (e.g., random vs. fixed effects for synthesis).

Review authors and Campbell coordinating groups are expected to adhere to, or oversee adherence to, these standards across all stages of the review process: protocols, reviews, and updates. Authors are required to provide a checklist affirming the adherence to the Campbell Standards to their respective editors when submitting the protocol and completed review to Campbell Systematic Reviews.

There are situations where parts of this guidance may not be applicable. First, when developing a protocol, some of the guidance related to analyzed data might not be relevant. Another instance is during reviews with qualitative syntheses. In such cases, authors should consult the latest Cochrane guidance found in the 2025 Campbell‐Cochrane Qualitative Evidence Synthesis Handbook and any subsequent updates. The Campbell Standards apply to all types of reviews; however, authors should refer to the Cochrane handbook (Chapter 12: Synthesizing and presenting findings using other methods) for specific guidance on selecting and justifying their methods. For reviews without meta‐analysis, authors must justify and detail how summary conclusions will be drawn from coded studies and report them according to the Synthesis without Meta‐analysis (SWiM) guidelines. We will continue to monitor methodological advances in synthesis approaches, especially as they relate to diverse study designs and update the standards and guidance as needed.

As the Campbell Standards represent broad best practice, individual review teams will likely need to use an additional reporting tool for more specific guidance on reporting each element of their review process. Thus, the reporting of reviews is expected to adhere to the relevant reporting guideline (e.g., PRISMA‐2020, and relevant extensions and transparently report which guideline was used (e.g., Meta‐analysis Of Observational Studies in Epidemiology [MOOSE], and Enhancing transparency in reporting the synthesis of qualitative research [ENTREQ]). All extensions can be found on the Enhancing the QUAlity and Transparency Of health Research (EQUATOR) Network. In addition, authors need to verify and ensure consistency of reporting results in the abstract, plain language summary, results and discussion.

We acknowledge that review methods will continue to develop, changing how they are being produced. The ongoing development of standards for qualitative evidence synthesis is an example where there is a need for future development and incorporation into these standards. We will continue to monitor the landscape and update these Campbell Standards as needed. We continue to welcome feedback from everyone involved in developing, using, or monitoring systematic review standards. Please provide your feedback in the *anonymous google form*. The working group will regularly review this feedback and, for transparency, will publish our responses to feedback on our OSF project page and any changes to the Campbell Standards. This new checklist aims to help Campbell produce better evidence for a better world (Table 1).

**Table 1:** Campbell Standards for Conduct and Reporting of systematic reviews, 2024.

Section of review	Page/paragraph no./line no.
**1. Scope of the review** Review questions need to be clear and address the issues important to interest holders such as consumers, policymakers, scholars, and others. Use an appropriate framework that captures the elements of your question (e.g., PICO for intervention reviews or PECO for exposure reviews) to develop the review question and objectives while particularly defining the outcomes of interest. If the review will address multiple interventions, clarify how these will be assessed (e.g., summarized separately, combined, or explicitly compared). The choice of synthesis approach should be justified to answer the question. Issues related to populations experiencing inequities and adverse effects should be considered as part of the review questions and objectives. Heterogeneity could impact the scope of the review. If heterogeneity is expected, the likely sources of heterogeneity should be defined, and subsequently, moderator analyses should be clearly described. Text describing a priori registration of protocol should be included. Deviations from the original questions outlined in the protocol should be justified and reported accordingly.	
(a) Formulate clear review questions	
(b) Indicate relevance to interest holders' needs	
(c) Consider issues related to populations experiencing inequities and how these will be considered	
(d) Discuss issues related to adverse effects and how these will be assessed	
(e) Pre‐define clear objectives including the objectives related to exploring heterogeneity or secondary questions	
(f) Justify synthesis approach to address the questions of the review	
**2. Eligibility criteria** Pre‐defined, unambiguous eligibility criteria are a fundamental prerequisite for a systematic review. Thus, it is important to construct eligibility criteria that align with the PICO (or alternative relevant framework for non‐intervention reviews) in the review questions and the objectives that other research teams can replicate. These eligibility criteria should be clear and well justified. The population could be rationally specified by setting, age, identifying personal characteristics, demographic factors, and other factors that differentiate the participants. Specification of comparator interventions requires particular clarity, including the extent to which the interventions are compared with a control or comparison conditions with matched or similar participants. Outcome measures are not necessarily part of the eligibility criteria of a review. However, some reviews legitimately restrict eligibility to specific outcomes. For example, the same intervention may be studied in the same population for different purposes, or a review may address specifically the adverse effects of an intervention used for several conditions. In such cases, the outcomes for this review should be explicitly mentioned. Study designs eligible for the review should be clearly defined with their features in addition to their labels and selected based on their appropriateness for the review. Studies should not be excluded on the basis of completeness of reporting of outcome data since this may introduce outcome reporting bias. Studies which meet eligibility criteria but have incomplete outcome data should be listed as included studies. If the review will exclude studies due to the risk of potential bias, there should be rationale for this decision as well as detailed information provided about how the review authors will determine and document the risk of bias during the eligibility review process.	
(g) Define population	
(h) Define intervention (or predictor)	
(i) Define comparator	
(j) Define primary and secondary outcomes	
(k) Define study designs	
(l) Define additional study factors (e.g., Time frame/geographical scope)	
**3. Search strategy** The goal of the search is to identify all eligible studies and to do so in a way that is transparent, replicable, and reduces the potential to further publication bias. To do this one needs to search multiple databases as well as gray literature and the reference lists of existing reviews and included studies. Additional methods, such as conducting a forward citation search of seminal works and included studies and hand searching key journals, can be used to improve comprehensiveness. The searches of databases should involve a well‐thought‐out set of keywords and make effective use of Boolean logic, wild cards, phrase searching, and subject headings, although one should be cautious of using some built‐in database filters. If possible, involve a librarian or information retrieval specialist with systematic review experience. A careful log of the search should be maintained and reported in the final review, including exact dates when the search was conducted, the results of the search, database and platform names, and the exact search strategy for each database. This includes all keywords, subject headings, and database syntax used for each bibliographic database and for gray literature resources when applicable. It is important to search for unpublished studies to mitigate the influence of reporting bias, including contacting experts in the field.	
(a) Describe search strategy in enough detail for replication (as described above)	
(b) Develop a search strategy that has a reasonable probability of identifying all studies that match eligibility criteria	
(c) Describe the reliability of the study inclusion process	
**4. Screening and Inclusion** The goal of screening studies for inclusion is to ensure that inclusion/exclusion decisions are transparent and reproducible. Screening is typically done in two stages. The first is a screening of the titles and abstracts obtained from the search strategy and the goal is to exclude studies which are obviously ineligible and identify those titles and abstracts that are potentially eligible and should be moved to the second stage. At the second stage, full texts of studies are screened for inclusion against the eligibility criteria. Throughout, the study team should maintain sufficient documentation for the creation of a PRISMA flowchart. Screening in duplicate will help reduce screening errors – that is, missing studies that should be included or erroneously including ineligible studies. The report should include a table of included and studies excluded at the full‐text screening (second stage). The list of excluded studies should list those studies which appear to meet inclusion criteria, that is, “near misses” and provide justification for exclusion. If automation is used (e.g., machine learning. AI screening of title and abstract), describe how, which software, including any validation (e.g., 10% review by human), if used.	
(a) Describe screening process and how it is designed to maximize reliability and replicability	
(b) Document the sources and flow of studies, with reasons for exclusion, using a PRISMA flowchart	
(c) Describe the reliability on the study inclusion process	
**5. Coding (also referred to as data extraction) and critical appraisal** The goal of coding (also referred to as data extraction) is to collect descriptive and statistical information in a fashion that is reliable and could be replicated by other researchers. The coding protocol should include both descriptive and statistical information needed for calculating effect sizes and produce needed tables (i.e., a table of study characteristics, risk‐of‐bias, etc.). Descriptive details should include details of the intervention, population, methods of outcome assessment, setting, and so forth. that would allow replication, as well as factors that might be associated with risks of bias such as funding, whether the study is led by the program developer, or other factors. The coding should be piloted on a sample of studies and refined if necessary. Information to inform critical appraisal is essential to collect as it is an integral component of high caliber evidence syntheses. Critical appraisal should be carried out with an appropriate risk of bias tool that includes assessment of domains such as selection bias, confounding, attrition that are appropriate for the study designs that are included. Additional information may be needed for planned or post hoc analyses, such as meta‐regression. Study coding should make use of a protocol with clearly defined variables and guidance on how to handle borderline cases (i.e., difficult coding decisions). The review needs to include processes for checking consistency across reviewers including piloting, consistency checking. When coding is conducted in duplicate, differences could be resolved either through consensus or by a third coder. When multiple manuscripts are available for a single study, use all available information for making coding decisions. It is best practice to reach out to authors for missing critical information about the study (e.g., such as information needed to calculate an effect size), and these efforts to contact authors should be documented, and ideally reach out to all authors with missing critical information. Choose and justify a critical appraisal tool that fits the purpose of the review.	
(a) Describe data coding and critical appraisal process (e.g., who and how many are involved, use of pre‐tested forms, machine learning, etc.) and how these are designed to minimize bias	
(b) Specify and provide rationale for each effect size index	
(c) Use all available manuscripts from the same study	
(d) Seek key information that is missing	
(e) Conduct critical appraisal assessment (e.g., assessment of risk of bias or study quality fit for the purpose of the review)	
**6. Synthesis methods** The synthesis methods should be well justified and use widely accepted analytical methods or established approaches as appropriate. Thus, this section should be adjusted according to the approach chosen and justified under Campbell Standard Sections 1 and 2. Describe the methods used to synthesize the results of the included studies, detailing whether a quantitative synthesis was planned. If a quantitative synthesis is not planned, clearly justify why and detail how summary conclusions will be drawn from coded studies. When conducting a quantitative synthesis, focusing solely on statistical significance is inappropriate. Explain how heterogeneity will be assessed and specify the chosen effect measure, such as standardized mean difference, odds ratio, risk ratio, correlation, or incident rate ratio. Describe how multiple effect sizes within studies will be handled, and outline the methods for meta‐analysis, whether a fixed‐effect or random‐effects model will be used. These methods should have been preplanned during the study planning stage. There are several estimators of the random effects variance component. The restricted maximum likelihood (REML) estimator is considered a good default but others are better suited to particular analytic situations. Heterogeneity affects the extent to which generalizable conclusions can be formed. It is important to identify heterogeneity in case there is sufficient information to explain it and offer new insights. Heterogeneity should be reported using appropriate metrics (e.g., Tau^2^, *I* ^2^, etc.). When quantitative effectiveness data has been collected, vote counting is never an accepted method of synthesis. If a quantitative synthesis is not planned, or if it is not possible, plan the specific methods to narratively synthesize the results of the included studies. Regardless of the synthesis approach chosen, justification must be provided for the choice, including addressing why the selected model/estimation method is appropriate for the analysis. Visualizations of the data, including forest plots should be used to present findings. There is overwhelming evidence of reporting biases of various types. Analyses of the results of included studies, for example using funnel plots or regression tests for funnel plot asymmetry, can sometimes help determine the possible extent of the problem, as can attempts to identify study protocols, which should be a more routine feature of a review.	
(a) Justify the choice of effect measure(s)	
(b) Justify choice of model and estimation method	
(c) Assess and report effect sizes, measures of variance, and heterogeneity	
(d) Use an appropriate method for handling dependencies among effect sizes	
(e) Examine heterogeneity if possible	
(f) Incorporate critical appraisal domains into moderator analysis, if possible	
(g) Explore publication and outcome selection bias	
**7. Discussion** The interpretation of results should focus on the pattern of evidence across studies and do so in light of (1) the quality of the evidence (i.e., critical appraisal), (2) the heterogeneity or variability in results across studies, and (3) the directness of the evidence to the review question. The directness of the evidence refers to the degree to which the intervention, context, population and settings align with the research question. Interpretation should discuss the meaningfulness of the size of effect in relation to the population and the population being studied. For example, an effect size for education is often benchmarked against the expected increase in years of learning. Interpretation should also consider certainty which includes the concepts of precision, inconsistency, risk of bias, including publication selection bias, and directness of the evidence. Interpretation should not be based on the statistical significance of the pooled effect. Review authors should discuss what new studies are needed to address weaknesses in the evidence base and what new directions in research we need to pursue to improve our understanding of the topic. Policy and practice recommendations are desirable but be cautious to not go beyond the evidence identified in the review. The “bottom line” of the review effort should be clearly communicated in the language appropriate for layman audiences through a layman abstract.	
(a) Interpret magnitude of effects in light of the credibility of the evidence (e.g., critical appraisal of evidence; publication/reporting bias)	
(b) Discuss uncertainty around key findings (i.e., heterogeneity of findings)	
(c) Interpret findings in light of the directness of the evidence (generalizability/external validity/construct validity)	
(d) Identify key implications for practice and research, to the extent justified from 7a, 7b, 7c	

