# Correction to “Campbell standards: Modernizing Campbell's Methodologic Expectations for Campbell Collaboration Intervention Reviews (MECCIR)”

**DOI:** 10.1002/cl2.70013

**Published:** 2024-12-12

Aloe, A. M., Dewidar, O., Hennessy, E. A., Pigott, T., Stewart, G., Welch, V., Wilson, D. B., & Campbell MECCIR Working Group. (2024) Campbell standards: Modernizing Campbell's Methodologic Expectations for Campbell Collaboration Intervention Reviews (MECCIR). *Campbell Systematic Reviews*, 20, e1445. Available from: https://doi.org/10.1002/cl2.1445

Below changes are made to the originally published article:

1. In the abstract, we invite readers to provide feedback on the article through a Google form. However, the link is not available. We intended to make “anonymous google form” as a hyperlink. We would like to add the following link: https://docs.google.com/forms/d/e/1FAIpQLSdPeuu0NqCGEojnqm6EZKlQpaJoNP74W17j4OuVS11dcvY‐jw/viewform
2. In Table 1, we list criteria using a, b, c, d, etc. under each section of the review. for example, under **1. Scope of the review**, we have (a) formulate clear review questions, (b) indicate relevance to interest holders' needs, etc. Under **2. Eligibility criteria**, it starts with (g) Define population and continues on with the remaining alphabetical order. Instead, we would like it to start with (a), (b), etc… to be consistent with the remaining sections.
3. In Table 1, we realized there is an error with Section **3. Search strateg**y (c) Describe the reliability of the study inclusion process. This should be **deleted** as it is a duplicate of a statement in Section 4.

We apologize for this error.

